# Epstein-Barr Virus Latent Membrane Protein-1 Induces the Expression of SUMO-1 and SUMO-2/3 in LMP1-positive Lymphomas and Cells

**DOI:** 10.1038/s41598-018-36312-4

**Published:** 2019-01-18

**Authors:** Sadia Salahuddin, Emma K. Fath, Natalie Biel, Ashley Ray, C. Randall Moss, Akash Patel, Sheetal Patel, Leslie Hilding, Matthew Varn, Tabithia Ross, Wyatt T. Cramblet, Angela Lowrey, Joseph S. Pagano, Julia Shackelford, Gretchen L. Bentz

**Affiliations:** 1 Departments of Medicine and Microbiology and Immunology, The University of North Carolina, Chapel Hill, NC USA; 2 Department of Cellular Biology and Physiology, The University of North Carolina, Chapel Hill, NC USA; 3 Lineberger Comprehensive Cancer Center, The University of North Carolina, Chapel Hill, NC USA; 4 Atta-ur-Rehman School of Applied Biosciences, National University of Sciences and Technology, Islamabad, Pakistan; 5 Department of Basic Medical Sciences, Mercer University School of Medicine, Macon, GA USA

## Abstract

Epstein-Barr Virus latent membrane protein-1 (LMP1) interacts with the SUMO-conjugating enzyme Ubc9, which induces protein sumoylation and may contribute to LMP1-mediated oncogenesis. After analyzing human lymphoma tissues and EBV-positive cell lines, we now document a strong correlation between LMP1 and *sumo-1/2/3* or SUMO-1/2/3 levels, and show that LMP1-induced *sumo* expression requires the activation of NF-κB signaling through CTAR1 and CTAR2. Together, these results point to a second mechanism by which LMP1 dysregulates sumoylation processes and adds EBV-associated lymphomas to the list of malignancies associated with increased SUMO expression.

## Introduction

Epstein-Barr virus (EBV) infects over 90% of the world’s population and establishes a life-long latent infection. EBV establishes distinct latency types (Type 0, I, II, and III) that are characterized by the expression profiles of the viral latency genes and the biologic properties of distinct lymphoid and epithelial malignancies^1,2^. The principal viral oncoprotein implicated in Type II latency (Hodgkin lymphoma and nasopharyngeal carcinoma) and in Type III latency (B-cell lymphomas in immunocompromised persons) is Latent Membrane Protein (LMP)-1^1,3,4^. LMP1 is an integral membrane signaling protein that mimics the tumor necrosis factor (TNF) receptor family members (such as CD40), with the exception that its activation is ligand independent and it is constitutively active^5^. As an oncoprotein, LMP1 significantly contributes to the sustained cellular proliferation and survival observed in EBV-associated malignancies. LMP1 consists of a short 24-amino-acid cytoplasmic N-terminal domain, six transmembrane domains (required for oligomerization of LMP1 and its constitutive activity), and a 200-amino-acid cytoplasmic C-terminal domain, which contains three C-terminal activating regions (CTARs)^5,6^. Most LMP1-induced signal transduction events are mediated through its extensively characterized C-terminal activating regions (CTAR)-1 and CTAR2^5,6^. However, we recently reported a novel function for the much less studied LMP1 CTAR3. We showed that LMP1 CTAR3 induces protein sumoylation via interaction with the SUMO-conjugating enzyme, Ubc9, during latent EBV infection^7,8^. In addition, we also reported that LMP1-induced protein sumoylation contributes to the maintenance of latent EBV infections^9^.

Protein sumoylation, a post-translational modification in which a small ubiquitin-like modifier (SUMO) is covalently attached to a lysine residue of a target protein, is a process very similar to protein ubiquitination^10,11^. Sumoylation processes are dynamic and reversible and can regulate protein function by altering a protein’s intracellular location, turnover, ability to interact with other proteins, or ability to interact with DNA^10,12,13^. Protein sumoylation is involved in central cellular processes, and multiple oncogene and tumor suppressor proteins undergo sumoylation, altering their function^14–19^. Furthermore, increases in protein sumoylation are a feature of a variety of types of cancer, including ovarian and colon cancer^20–26^. Because cellular sumoylation processes are thought to be critical in regulating oncogenesis, elements of the sumoylation process have been proposed as new targets for cancer therapies^22,27^.

Sumoylation processes have a role in the EBV life-cycle^11,28–36^. We documented that LMP1 CTAR3 physically interacts with functional Ubc9 during latent EBV infections^8^, and increases sumoylation of cellular proteins, including interferon regulatory factor-7 (IRF7)^7^ and KRAB-associated protein-1 (KAP-1)^9^. The LMP1-Ubc9 interaction contributes to basic features of the oncogenic phenotype produced by LMP1^8^. These results led us to ask whether LMP1 can dysregulate cellular sumoylation processes by additional mechanisms. Because increases in levels of SUMO-1 have been detected in several malignancies^20–26^, we were specifically interested in whether LMP1 induced the expression of *sumo-1* and *sumo-2/3*, which could provide increased pools of SUMO proteins available for the modification of cellular target proteins.

Banked gene-array data were analyzed to examine *sumo* levels in EBV-associated malignancies. In three studies, nasopharyngeal carcinoma tissues and established nasopharyngeal carcinoma lines expressed increased *sumo* levels compared with normal nasopharyngeal tissues (data set records GDS3341, GSE34573, and GDS3610)^37–39^. A fourth study documented that both non-Hodgkin and Hodgkin lymphoma tissues expressed higher *sumo* levels than normal B-cells (GDS3516)^40^. Because these studies strengthened our implication that *sumo* levels are increased in EBV-associated malignancies, we examined gene-array data in which EBV status was considered. In two studies examined, EBV-positive B-cells expressed higher levels of *sumo* RNA than their EBV-negative counterparts (GSE45919 and GDS1063)^41,42^. Together these studies led us to propose a second mechanism by which LMP1 dysregulates cellular sumoylation processes; namely, by inducing the expression of *sumo-1/2/3*.

We show here that there was a strong correlation between LMP1 and *sumo-1/2/3* or SUMO-1/2/3 levels in EBV-positive cell lines and EBV-positive lymphomas. LMP1 is necessary and sufficient to induced *sumo* expression, and this induction requires the activation of NF-κB signaling through CTAR1 and CTAR2. These results identify a second mechanism by which LMP1 dysregulates sumoylation processes during latent EBV infection.

## Materials and Methods

### Cells

HEK 293 cells were maintained in Dulbecco’s modified Eagle’s medium (DMEM) plus 10% fetal bovine serum (FBS). BL41 EBV-negative cells, BL41 EBV WT cells, BL41 EBV P3HR1 cells^43–45^, and EBV-positive lymphoblastoid cell lines (KR4, LCL1, LCL2, LCL3) were maintained in RPMI with 10% FBS. Two KR4-HeLa fusion cell lines (KH1 and KH2) were maintained in RPMI with 10% FBS. Additionally, three EBV-infected human breast cancer cell lines (MDA-MB-231 C3B4, C3G6, C4A2, which express high, moderate, and low/undetectable levels of LMP1, respectively^46–48^) were maintained in RPMI with 10% FBS and 700 μg/ml G418 (Cellgro). Peripheral B-cells were isolated from Human Whole Peripheral Blood (purchased from StemCell Technologies) using RosetteSep™, Lymphoprep™, and RosetteSep™ Human B cell Enrichment Cocktail (StemCell Technologies).

### Transient Transfection

FLAG-LMP1- and select mutant FLAG-LMP1-expresion constructs have been described previously^7,8^. Briefly, 293 cells were grown in 100-mm dishes, serum-starved for 24 hours (DMEM lacking FBS), and transfected with plasmid expression constructs using PEI. Typically, 1 μg of any LMP1-expression construct was used per 100 mm dish; however, dose-dependent experiments used a range of 0–5 μg of plasmid DNA per 100 mm dish. 24 hours post-transfection, cells were collected for whole-cell lysates and RNA.

### Mitogen Blasts

Purified B-cells were stimulated with LPS (20 μg/ml; Sigma-Aldrich)^49,50^. After 48 hours of stimulation, cells were collected and RNA harvested.

### Reverse Transcription and Real-time PCR

RNA was harvested with the use of the Qiagen RNeasy Plus Mini Kit and reverse-transcribed with the Applied Biosystems High Capacity cDNA Reverse Transcription Kit. cDNA were analyzed by real-time PCR with the ABI 7900HT and A7300 system. iTaq SybrGreen Universal Master Mix (Bio-Rad) was used with the following primers: *gapdh* (5′ TCATCAGCAATGCCTCCT 3′; 5′ AGGGGCCATCCACAGTCTTC 3′), *sumo-1* (5′ TCAAAGACAGGGTGTTCC 3′; 5′ CCCCGTTTGTTCCTGATA 3′), LMP1 (5′ AGGTTGAAAACAAAGGAGGTGACCA 3′; 5′ GGAACCAGAAGAACCCAAAAGCA 3′), and *sumo-2/3* (5′ AGCCCAAGGAAGGAGTGAAG 3′; 5′ TTGACAATCCCTGTCGTTCA 3′). Reactions and experiments were performed in triplicate, and relative RNA levels (relative to *gapdh*) or fold change in relative RNA levels (relative to gapdh) was determined.

### Slot-blots and Western blot analyses

Whole-cell lysates were denatured in 4x SDS loading buffer and boiled for 10 minutes. Slot-blot analyses, where lysates were collected on nitrocellulose membranes (Bio-Rad), were performed to examine total protein levels^51^. Whole-cell lysates were also separated by 4–20% SDS-polyacrylamide gradient gel electrophoresis (SDS-PAGE) and transferred to polyvinylidene fluoride membranes (PVDF; Bio-Rad). Membranes were blocked with 5% milk in Tris-buffered saline-Tween-20 (TBST) and incubated overnight at 4 °C with primary antibodies. The membranes were washed and incubated with appropriate horseradish peroxidase-conjugated secondary antibodies for one hour at room temperature. Membranes were washed again, and bands were visualized with enhanced chemiluminescence reagent from Advansta.

### Lymphoma Samples

Anonymous samples were collected from patients following informed consent in hospitals of Islamabad and Faisalabad, Pakistan during the 1990′s. The samples selected for the studies were determined as B-cell lymphomas (DLBCL) and Hodgkin’s Lymphoma (HL) by Armed Forces Institute of Pathology (AFIP), Rawalpindi. This study was approved by the Institutional Review Board (IRB) of Atta-ur-Rahman School of Applied Biosciences, National University of Sciences and Technology, Islamabad and was performed in accordance with the principles expressed in the Declaration of Helsinki.

### RNA Extraction from Lymphoma Samples

42 formalin-fixed paraffin-embedded tissue samples (FFPET blocks) of multiple B-cell lymphomas were selected and RNA was extracted (High Pure RNA Paraffin Kit; Roche). RNA was reverse-transcribed with the Applied Biosystems High Capacity cDNA Reverse Transcription Kit, and real-time PCR was performed for *gapdh*, *sumo-1*, and *sumo-2/3* (primers described above). Reactions for the target and reference genes were performed in duplicate, and relative *sumo-1*, *sumo-2/3*, and LMP1 levels (relative to *gapdh*) were determined.

### Immunofluorescence Microscopy

LMP1-negative and LMP1-positive lymphomas FFPET blocks were sectioned (4-μm), fixed on glass slides, permeabilized with 0.1% Triton-X100, blocked with normal donkey serum, and stained with LMP1-specific (S12 and CS1–4; AbCam) and SUMO-1-specific (FL-101; Santa Cruz Biotechnologies) antibodies overnight. After washing with PBS, slides were incubated with appropriate secondary antibodies (goat anti-mouse IgG Highly Cross-Adsorbed Secondary Antibody, Alexa Fluor 488 or goat anti-rabbit Highly Cross-Adsorbed Secondary Antibody, Alexa Fluor 594) for one hour. Following additional rinses, coverslips were mounted (ProLong® Gold Antifade Reagent with DAPI; Invitrogen) onto slides, immunofluorescence microscopy was performed (20X magnification), and images created using Openlab software (Improvision Inc.)

HEK 293 cells were grown on glass coverslips, serum-starved, and transfected with control- and LMP1-expression constructs. 24 hours post-transfection, cells were fixed in 4% paraformaldehyde, permeabilized with 0.1% Triton-X100, blocked with normal donkey serum, and stained with FLAG-specific (M2 Sigma) and SUMO-1-specific (FL-101; Santa Cruz Biotechnologies) antibodies overnight. After washing with PBS, slides were incubated with appropriate secondary antibodies (goat anti-mouse IgG Highly Cross-Adsorbed Secondary Antibody, Alexa Fluor 488 or goat anti-rabbit Highly Cross-Adsorbed Secondary Antibody, Alexa Fluor 568) for one hour. Following three additional rinses, coverslips were mounted (ProLong® Gold Antifade Reagent with DAPI; Invitrogen) onto slides, and microscopy was performed at 60X magnification using the Nikon A1 laser confocal microscope.

### Antibodies and Drugs

Mouse Anti-FLAG (M2) antibodies were purchased from Sigma. Rabbit Anti-SUMO-1 and anti-SUMO-2/3 antibodies were obtained from Cell Signaling. Mouse Anti-SUMO-1 (9E10) and rabbit anti-GAPDH (FL-335) antibodies, Bay 11–7082 and LY294002 were purchased from Santa Cruz Biotechnologies. Mouse Anti-LMP1 antibodies (CS 1–4) were purchased from Dako or Abcam. Horseradish peroxidase-conjugated Goat anti-mouse and goat anti-rabbit secondary antibodies were purchased from Bio-Rad.

### Statistical Analysis

Statistical analyses were performed using unpaired, two-tailed, Student’s T-test. Unless indicated, data are presented as means ± the standard deviation for samples run in triplicate and independent experiments performed in triplicate. Differences were considered statistically significant when P-values were less than 0.05.

## Results

### *sumo-1/2/3* and SUMO-1/2/3 levels increased as LMP1 levels increased in EBV-positive cell lines

**Figure 1:**
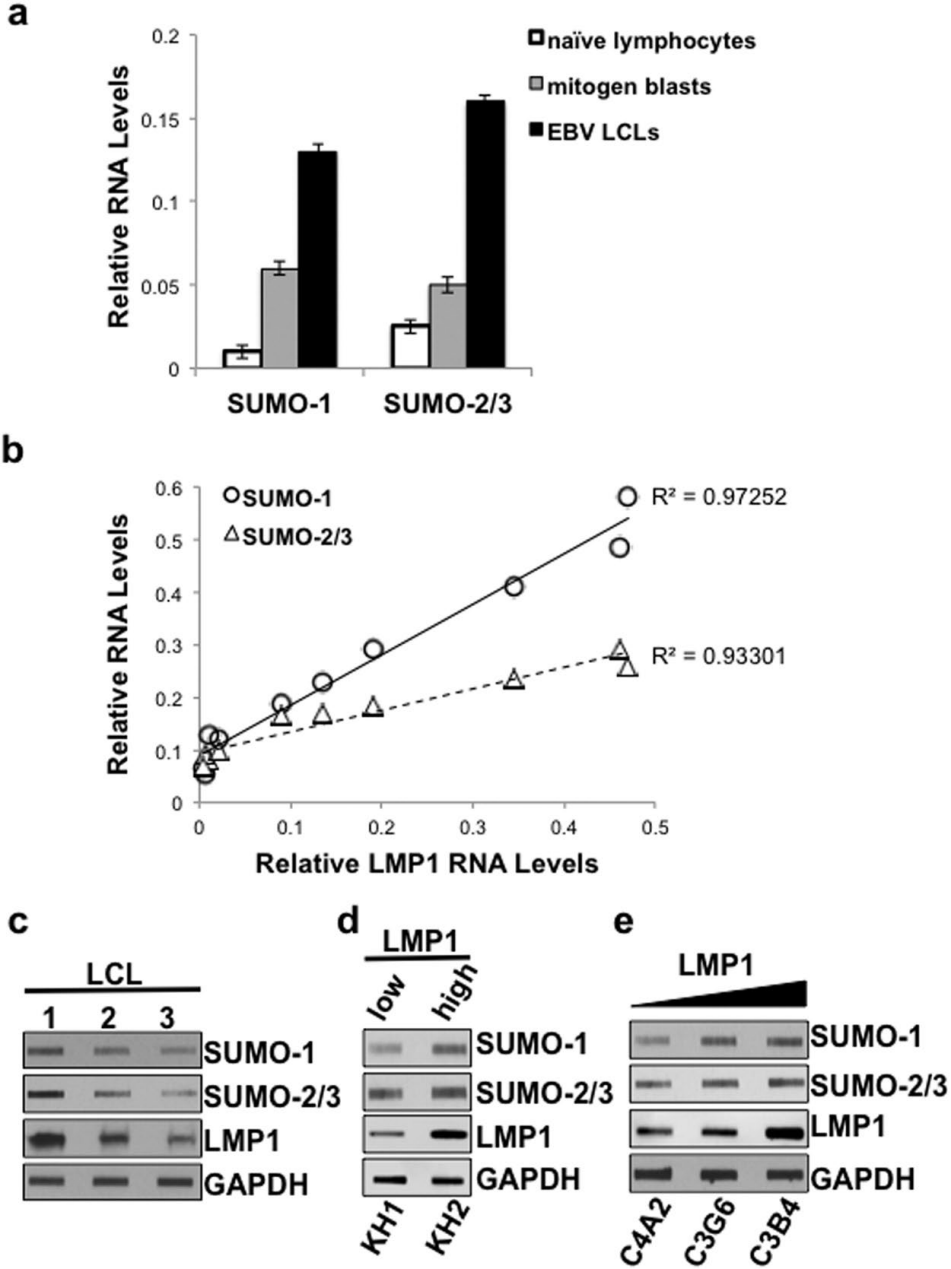
*sumo-1/2/3* and SUMO-1/2/3 levels increased as LMP1 levels increased in EBV-positive cell lines. (**a**) RNA was extracted from paired naïve lymphocytes, mitogen blasts, and EBV-transformed LCLs. cDNAs were generated, and real-time PCR was performed and relative *sumo-1*, and *sumo-2/3* levels (relative to *gapdh*) determined. Results are shown as the mean ± the standard deviation for samples performed in triplicate. Independent experiments were performed in triplicate. (**b**–**e**) RNA and protein were harvested from ten different EBV-positive cell lines. (**c**) cDNAs were generated, and real-time PCR was performed to quantitate relative LMP1, *sumo-1*, and *sumo-2/3* levels (relative to *gapdh*). Results are shown as the mean for samples performed in triplicate from three independent experiments. Regression analysis of LMP1 and *sumo-1* or *sumo-2/3* levels was performed. (**d**–**f**) Slot immunoblots were performed to detect LMP1, SUMO-1, and SUMO-2/3 levels in (**d**) three EBV-transformed LCLs, (**e**) two KR4-HeLa fusion cell lines, and (**f**) three EBV-infected human breast cancer cell lines. GAPDH was used as a loading control. Representative blots for experiments performed in triplicate are shown.

To begin to test our hypothesis that LMP1 induces the expression of *sumo-1/2/3*, we analyzed *sumo* and LMP1 RNA levels (relative to *gapdh*) in naïve human lymphocytes and their mitogen-activated or EBV-transformed lymphoblastoid cell line (LCL) counterparts^49,50^. As expected, LMP1 RNA levels were only detected in EBV-transformed LCLs, with no LMP1 detected in naïve B-cells or the mitogen blasts (data not shown). In addition, while the mitogen blasts expressed increased *sumo-*1 and *sumo-2/*3 levels when compared with naïve B cells (Fig. 1a), significant (P < 0.05) increases in *sumo-1* and *sumo-2/3* levels were also detected in the EBV-transformed LCLs (Fig. 1a). These data suggested that *sumo* levels were increased following EBV-mediated transformation and support our analysis of previous gene-array data^41,42^. The findings led us to investigate further the role of LMP1 in the induction of *sumo-1/2/3* levels during EBV latency.

Using real-time PCR, LMP1 and *sumo-1/2/3* levels were examined in five established EBV-positive LCLs (BL41 EBV-positive, KR4, LCL1, LCL2, LCL3: represent Type III latency), two KR4-HeLa fusion cell lines (KH1 and KH2: represent Type II latency), and three EBV-infected human breast cancer cell lines (MDA-MB-231 C3B4, C3G6, C4A2, which express high, moderate, and low/undetectable levels of LMP1, respectively^46–48^). Results revealed a strong, positive correlation (R^2^ >0.9) between endogenous LMP1 and endogenous *sumo-1* or *sumo-2/3* levels (Fig. 1b). The KR4-HeLa fusion cell lines and EBV-infected human breast cancer cell lines expressed much lower LMP1 levels than the EBV-positive LCLs; however, they also had lower *sumo* levels than the EBV-positive B-cells. Similar correlations were observed following analyses of EBV-positive LCLs alone or EBV-positive adherent cells alone (data not shown).

Slot-blot analyses were performed on whole-cell lysates to determine if the changes observed at the RNA levels corresponded with changes at the protein level^51^. Comparison of lysates from three LCLs (Fig. 1c), KH1 and KH2 cells (low and high LMP1 expression, respectively; Fig. 1d), and MDA-MB-231 C3B4, C3G6, and C4A2 cells (Fig. 1e) revealed that SUMO-1 and SUMO-2/3 levels paralleled LMP1 levels. Together, these data demonstrated a consistent correlation between LMP1 and *sumo-1/2/3* levels during latent EBV infection. In addition, the increased *sumo* levels correspond with increased protein levels. The findings led us to hypothesize that LMP1 induces the expression of *sumo*, which results in increased intracellular SUMO pools and contributes to enhanced protein sumoylation during EBV latency.

### LMP1 was necessary and sufficient to increase *sumo-1/2/3* and SUMO-1/2/3 levels *in vivo*

**Figure 2:**
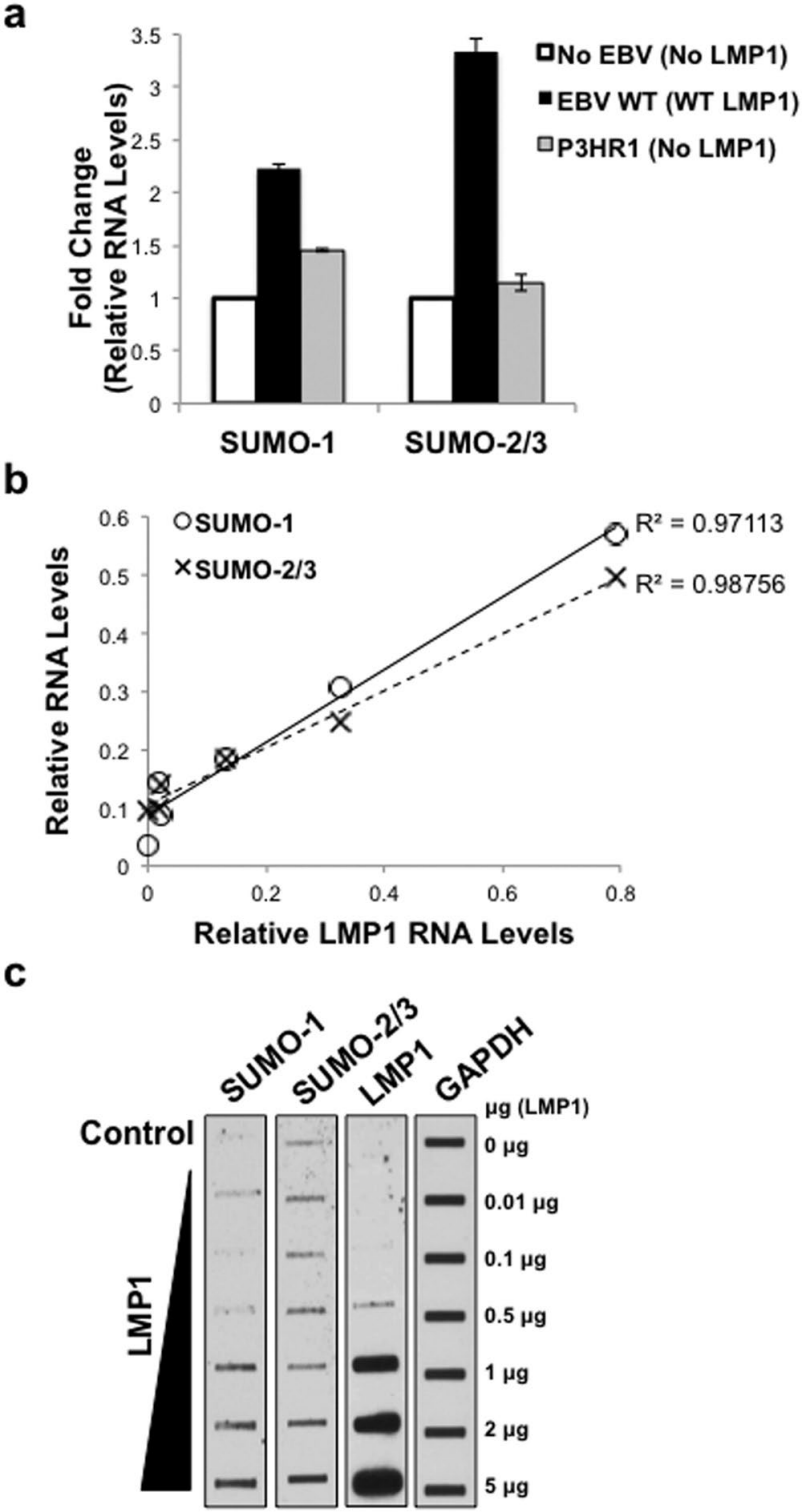
LMP1 was necessary and sufficient to increase *sumo-1/2/3* and SUMO-1/2/3 levels *in vivo*. (**a**) Real-time PCR was performed using cDNA generated from RNA harvested from BL41 EBV-negative, BL41 EBV WT, and BL41 EBV mut (P3HR1) cells. The fold change in relative *sumo-*1 and *sumo-*2/3 expression (relative to *gapdh*) was determined. Results are shown as the mean ± the standard deviation samples run in triplicate and independent experiments performed in triplicate. (**b**–**c**) 293 cells were serum-starved for 24 hours and then transfected with graduated amounts of an LMP1-expression vector or a control vector in serum-free media. (**b**) RNA was harvested, cDNA prepared, and real-time PCR performed. Relative LMP1, *sumo-1*, and *sumo-2/3* levels were determined (relative to *gapdh*). Regression analysis of LMP1 and *sumo-1* or *sumo-2/3* levels was performed. Samples were run in triplicate and independent experiments performed in triplicate. (**c**) Slot blots were performed to detect SUMO-1, SUMO-2/3, and LMP1 levels. GAPDH was used as a loading control. Representative blots for experiments performed in triplicate are shown.

The importance of LMP1 in the observed induction of *sumo-1* and *sumo-2/3* was verified in a set of paired cell lines (BL41 EBV-negative cells lack both virus and LMP1; BL41 EBV WT cells are virus-infected and express WT LMP1; and BL41 EBV mut (P3HR1) cells are virus-infected yet there is no detectable LMP1 expression due to the deletion of EBNA2^43–45^). BL41 EBV WT cells expressed over twice as much *sumo-1* and *sumo-2/3* than BL41 EBV-negative and BL41 EBV mut cells (Fig. 2a). Surprisingly, while *sumo-1/2/3* levels in BL41 EBV mut cells were significantly (P < 0.05) less compared with BL41 EBV-positive cells, expression levels were still significantly (P < 0.05) greater when compared with BL41 EBV-negative cells.

The correlation between *LMP1* and *sumo-1* or *sumo-2/3* expression was further confirmed following transient transfection of graduated amounts of LMP1-expression constructs^7,8^ into serum-starved HEK 293 cells. Results showed a strong, positive correlation (R^2^ > 0.9) between LMP1 and *sumo-1/2/*3 levels (Fig. 2b). Slot-blot analyses of whole-cell lysates from transfected cells revealed that SUMO-1/2/3 levels increased as LMP1 levels increased (Fig. 2c). These findings documented that LMP1 was necessary and sufficient for the induction of *sumo-1/2/3* and SUMO-1/2/3 levels.

### LMP1 CTAR1 and CTAR2 contributed to LMP1-mediated increase in *sumo* levels via the activation of NF-κB

**Figure 3:**
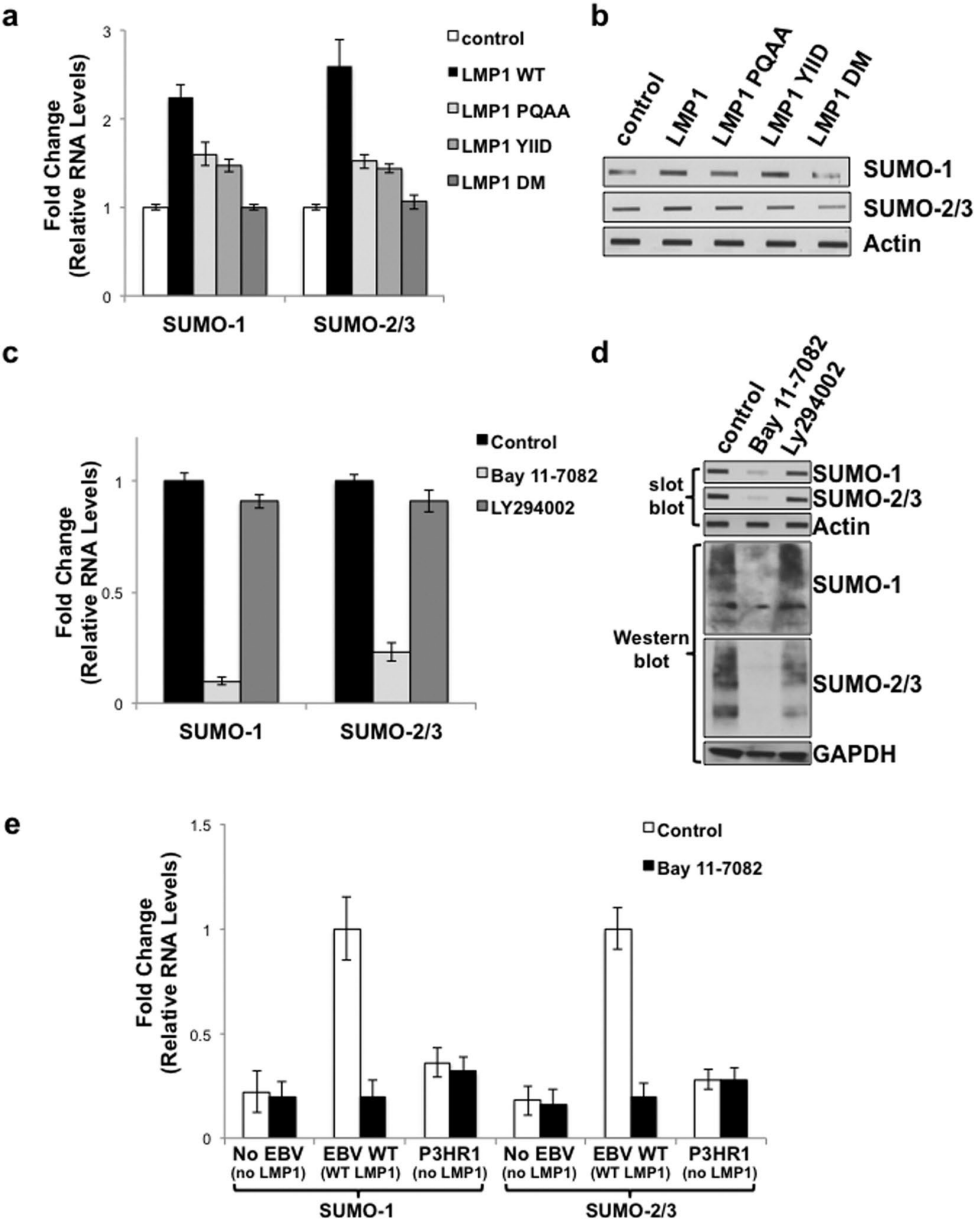
LMP1 CTAR1 and CTAR2 contributed to LMP1-mediated increase in *sumo* levels via the activation of NF-κB. (**a**,**b**) 293 cells were transfected with 1 μg of a LMP1-expression plasmid, select LMP1 mutant-expression plasmid, or a pcDNA3 control plasmid. (**a**) RNA was harvested and cDNA was prepared. Real-time PCR was performed to quantitate relative *sumo-1* and *sumo-2/3* levels (relative to *gapdh*), The fold change in *sumo-*1 and *sumo-2/3* levels (compared to control-expressing cells) was determined. Results are shown as the mean ± the standard deviation for samples performed in triplicate and independent experiments performed in triplicate. (**b**) Slot blots were performed to detect SUMO-1 and SUMO-2/3 levels. Actin was used as a loading control. Representative blots for experiments performed in triplicate are shown. (**c**,**d**) EBV-transformed LCLs were treated with Bay 11–7082 (1 μM), LY294002 (5 μM), or DMSO (the vehicle control; Control) for 24 hours. (**c**) RNA was harvested and cDNA was prepared. Real-time PCR was performed to quantitate relative *sumo-1*, and *sumo-2/3* expression (relative to *gapdh*), and the fold change in *sumo-1* and *sumo-2/3* levels (relative to control-treated cells) was determined. Results are shown as the mean ± the standard deviation for samples performed in triplicate and independent experiments performed in triplicate. (**d**) Slot immunoblots and Western blots analyses were performed to detect SUMO-1 and SUMO-2/3 levels. GAPDH was used as a loading control. Representative blots for experiments performed in triplicate are shown. (**e**) cDNA generated from RNA harvested from BL41 EBV-negative, BL41 EBV WT, and BL41 EBV mut (P3HR1) cells that were treated with DMSO (the vehicle control; control) or Bay 11–7085 (1 μM) for 24 hours. Real-time PCR was performed was performed to quantitate relative *sumo-1*, and *sumo-2/3* levels (relative to *gapdh*). The fold change in *sumo-1* and *sumo-2/3* levels (relative to control-treated BL41 EBV WT cells) was determined. Results are shown as the mean ± the standard deviation for samples performed in triplicate and independent experiments performed in triplicate.

Next, we investigated the requirements for LMP1-mediated induction of *sumo-1/2/3* using select LMP1 mutants over-expressed in serum-starved HEK 293 cells^52^. Results showed that mutation of CTAR1 (LMP1 PQAA) or CTAR2 (LMP1 YIID) significantly (P < 0.05) inhibited LMP1-induced *sumo-1* and *sumo-2/3* levels (Fig. 3a). Mutation of both CTAR1 and CTAR2 (LMP1 DM) returned *sumo-1* and *sumo-2/3* levels to those detected in control cells. While somewhat difficult to detect due to the transformed nature of the HEK 293 cells, levels of SUMO-1 and SUMO-2/3 seemed to correlate with their respective RNA levels (Fig. 3b). These findings suggest that CTAR1 and CTAR2 both contribute to the activation of the *sumo* promoters resulting in increased protein levels.

While LMP1 CTAR1 and CTAR2 activate multiple signaling pathways^5,6^, both activating regions have been implicated in the activation of IκB-dependent canonical NF-κB signaling^53,54^, which regulates the expression of genes involved in proliferation, metastasis, and cell death^55,56^ and is critical for the survival of EBV-transformed LCLs^57,58^. To test if LMP1 induces *sumo-1/2/3* expression through NF-κB, EBV-transformed LCLs were treated with Bay 11–7082 (1 μM), which inhibits the phosphorylation of IκBα. As a control, cells were treated with DMSO (the vehicle control) or with the phosphatidylinositol 3-kinase (PI3K) inhibitor LY294002 (5 μM; Santa Cruz Biotechnologies), in light of the findings that LMP1 activates PI3K signaling via CTAR1^59–61^. Findings revealed that LCLs treated with Bay 11–7082 expressed significantly lower *sumo-1* and *sumo-2/3* levels (P < 0.05) when compared with DMSO-treated cells (Fig. 3c). Treatment of cells with LY294002 did not significantly alter *sumo-1* or *sumo-2/3* levels. Total SUMO-1 and SUMO-2/3 levels correlated with their respective RNA levels (Fig. 3d), which suggested that LMP1 induces *sumo-1/2/3* and increases SUMO-1/2/3 levels in a NF-κB-dependent manner.

To determine if the Bay 11–7085-mediated inhibition of *sumo* levels was dependent on LMP1 expression, the BL41 paired cell lines^43–45^ were treated with Bay 11–7082 (1 μM) or DMSO (the vehicle control). Data confirmed that BL41 EBV WT cells expressed higher *sumo-1* and *sumo-2/3* levels than BL41 EBV-negative and BL41 EBV mut cells (Fig. 3e); however, Bay 11–7082 only decreased *sumo-1* and *sumo-2/3* levels in the WT-LMP1-expressing cells (Fig. 3e). These data further supported our proposal that LMP1 induces *sumo-1/2/3* and increases SUMO-1/2/3 levels in a NF-κB-dependent manner.

### Increased *sumo-1/2/3* and SUMO-1 levels were detected in LMP1-positive lymphoma tissues in a LMP1-dependent manner

**Figure 4:**
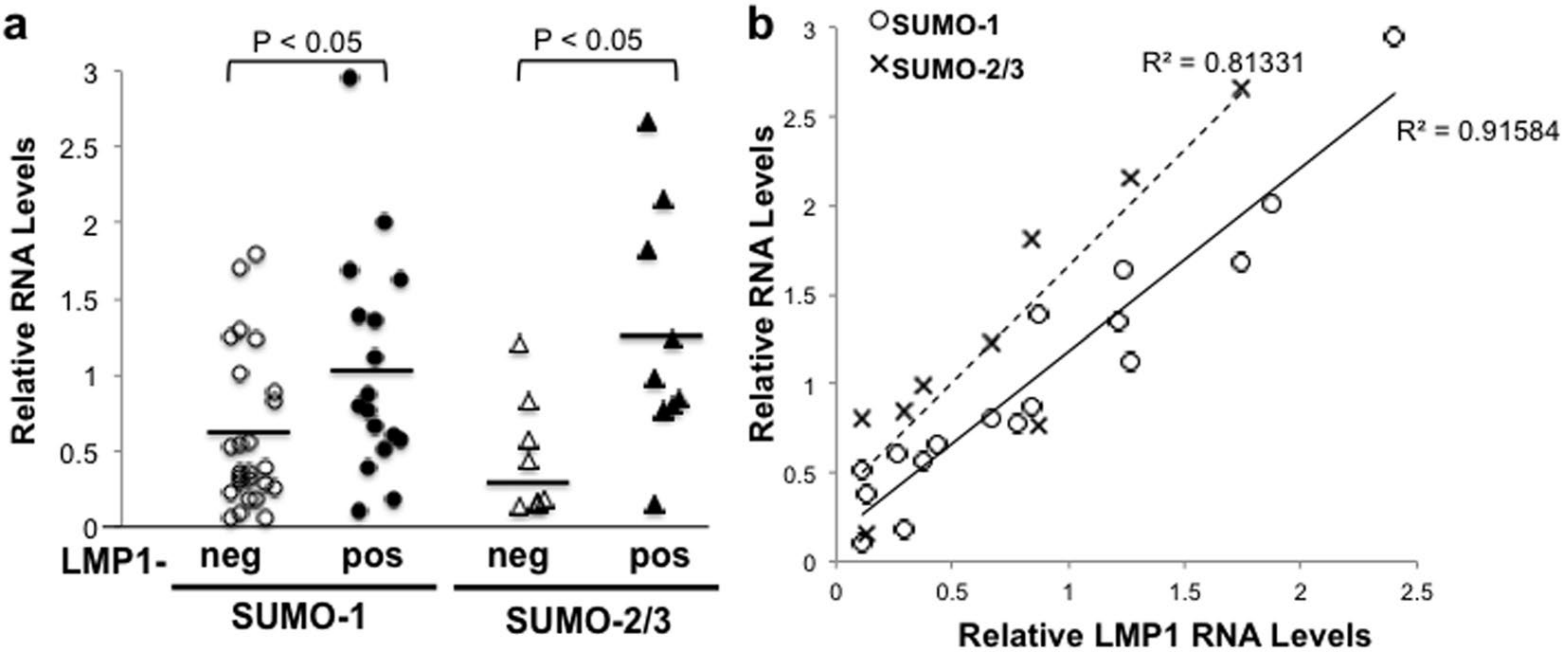
Increased *sumo-1/2/3* levels were detected in LMP1-positive lymphoma tissues in an LMP1-dependent manner. (**a**,**b**) RNA was extracted from 42 biopsy tissues and cDNA was made. Real-time PCR was performed to quantitate *gapdh*, LMP1, and *sumo-1* levels. RNA yields allowed *sumo-2/3* levels to be tested in 17 of the 42 samples. (**a**) Relative LMP1, *sumo-1* and *sumo-2/3* levels (relative to *gapdh*) were determined. The detection of LMP1 RNA levels determined is tissues were LMP1-negative (LMP1-neg) and LMP1-positive (LMP1-pos). Results are shown as the mean relative *sumo-1* and *sumo-2/3* levels for individual samples (shapes). Horizontal lines represent man *sumo-1* and *sumo-2/2* levels for the collective LMP1-neg and LMP1-pos samples. (**b**) Of the 19 LMP1-positive tissue samples, relative *sumo-*1 levels were determined in all 19 samples but relative *sumo-2/*3 levels were determined in only 9 samples. Results are shown as the mean relative LMP1, *sumo-*1, and *sumo-2/*3 leves of samples run in duplicate. Regression analysis was performed.

Although the correlation between LMP1 and *sumo-1/2/3* levels was observed in multiple cell lines and following transfection of HEK 293 cells, the question remains if there is a similar association *in vivo*, specifically in lymphomas. We analyzed 42 formalin-fixed paraffin-embedded tissue samples (FFPET blocks) of multiple B-cell lymphomas obtained from pathologists in selected hospitals in Pakistan. While the pathologists did classify each sample as a Hodgkin’s and non-Hodgkin’s lymphoma, the tissue samples were not analyzed for the presence or absence of EBV. Results showed that LMP1 was detected in 19 of the 42 tissues (Fig. 4a). Mean relative *sumo-1* levels were significantly higher (P < 0.05) in LMP1-positive lymphoma tissues compared with LMP1-negative lymphoma tissues. Due to limited tissue RNA yields from some of the fixed tissue, relative *sumo-2/3* levels were determined in 17 of the 42 original samples, and nine of these 17 samples were positive for LMP1 (Fig. 4a). LMP1-positive tissues contained significantly (P < 0.01) higher *sumo-2/3* levels than LMP1-negative tissues (Fig. 4a). These data are the first to demonstrate increased *sumo-1/2/3* levels in EBV-associated and LMP1-positive lymphomas.

To further investigate the relation between expression of LMP1 and SUMO-1, correlative analyses at the RNA level were determined in the lymphoma tissues. Regression analyses revealed a strong, positive correlation (R^2^ > 0.8) between relative LMP1 and *sumo-1* or *sumo-2/*3 RNA levels (Fig. 4b), which demonstrated that *sumo* levels were increased in EBV-positive lymphomas in an LMP1-dependent manner.

**Figure 5:**
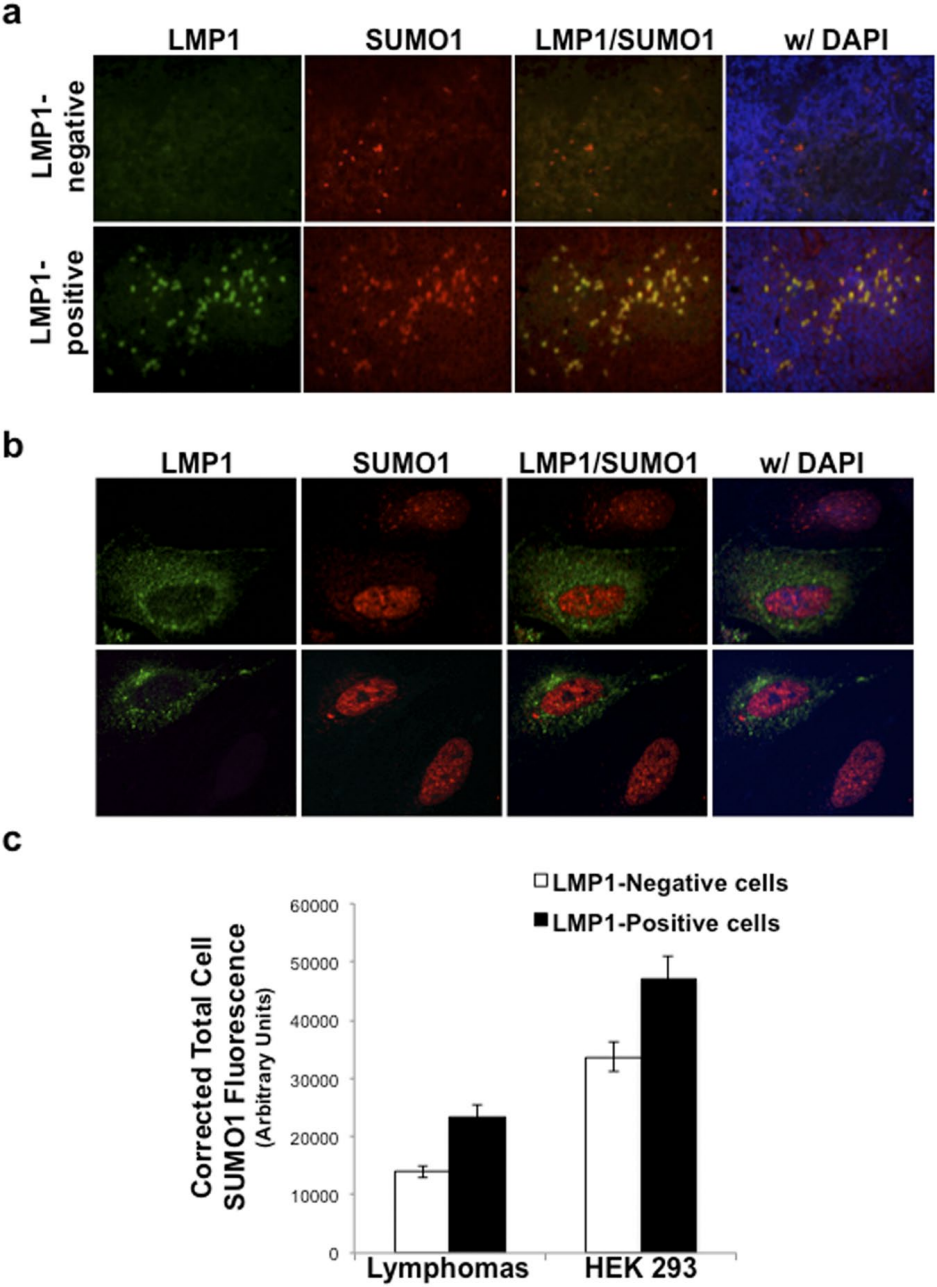
Increased SUMO levels were detected in LMP1-positive lymphoma tissues and cells. (**a**) FFPET biopsy tissues were sectioned onto glass slides. Following staining with rabbit anti-SUMO-1-specific and mouse anti-LMP1-specific primary antibodies and goat anti-mouse Alexa Fluor 488 or goat anti-rabbit Alexa Fluor 594 secondary antibodies, coverslips were mounted using ProLong® Gold Antifade Reagent with DAPI. Immunofluorescence microscopy was performed at 20X magnification and images created using the Openlab software. (**b**) Serum-starved 293 cells were transfected with FLAG-LMP1-expression constructs. 24 hours post-transfection, cells were fixed, permeabilized, and stained with rabbit anti-SUMO-1-specific and mouse anti-FLAG-specific primary antibodies and goat anti-mouse Alexa Fluor 488 or goat anti-rabbit Alexa Fluor 594 secondary antibodies. Coverslips were mounted using ProLong® Gold Antifade Reagent with DAPI. Confocal microscopy was performed at 60X magnification using the Nikon A1 laser confocal microscope. Images of LMP1-transfected cells next to non-LMP1-expressing cells were captured. Representative images are shown. (**c**) The corrected total cell fluorescence was determined for SUMO-1 levels in approximately 150 LMP1-negative and 150 LMP1-positive cells in lymphoma tissue samples and approximately 65 LMP1-negative and 65 LMP1-positive 293 cells. Results are shown as the mean ± the standard deviation.

Immunofluorescence microscopy was performed on LMP1-negative and LMP1-positive lymphomas in order to determine if increased *sumo* RNA levels correlated with increased SUMO protein levels in the tissue samples (Fig. 5a). LMP1 expression was readily detectable in lymphoma cells within the LMP1-positive tissue samples, but not in the LMP1-negative tissue samples. As expected, SUMO-1 expression was detected in all cells in each tissue sample. The LMP1-negative lymphoma tissue sample had few cells with increased SUMO levels when compared with the surrounding cells. The LMP1-positive lymphoma tissues samples contained more numerous cells with increased SUMO-1 levels than the LMP1-negative sample. LMP1 was detected in the majority of cells exhibiting increased SUMO-1 levels, supporting our claim that LMP1 expression resulted in increased expression of SUMO-1. These data were confirmed in serum -starved 293 cells transfected with an LMP1-expression construct. Images were captured where LMP1-positive and LMP1-negative 293 cells were visible in the same field of view. LMP1-expressing cells exhibited increased SUMO-1/2/3 levels when compared with LMP1-negative cells (Fig. 5b). In most cases, increased SUMO levels detected in the nuclei of LMP1-expressing cells. In some instances, increased SUMO levels occurred throughout the nucleus, but some LMP1-expressing cells displayed increased SUMO-positive clusters, which may be due to LMP1-induced increased formation of promyelocytic leukemia protein nuclear bodies^62,63^. Interestingly, all LMP1-expressing cells also exhibited increased cytoplasmic staining of SUMO-1/2/3 when compared with their non-LMP1-expressing counterparts. The corrected total cell SUMO1 fluorescence was determined for LMP1-positive and LMP1-negative cells in the tissue samples and following transfections^64–67^, and results showed that LMP1-positive cells had significantly (P < 0.05) higher SUMO1 levels than their LMP1-negative counterparts (Fig. 5c). These findings confirm that LMP1 expression increased total cellular SUMO levels, and while most SUMO localized to the nucleus, we now show that LMP1 expression increases the levels of cytoplasmic SUMO.

Together, these results demonstrate that *sumo*, and SUMO, levels are increased in EBV-associated malignancies, and suggest that this induction occurs in an LMP1-dependent manner due to the activation of NF-κB by CTAR1 and CTAR2. The data identify a novel role for CTAR1 and CTAR2 in the regulation of protein sumoylation. Because we previously documented that LMP1 induces the sumoylation of cellular proteins by interacting with Ubc9 via CTAR3^8^, these findings document a second mechanism by which LMP1 dysregulates cellular sumoylation processes and suggest that LMP1 regulates sumoylation processes by a multi-pronged approach.

## Discussion

Previously, we documented that LMP1 induces protein sumoylation *in vitro* following transient transfection of HEK 293 cells^8^. Specifically, results show that LMP1 interacts with Ubc9, through CTAR3, to increase the sumoylation of cellular proteins^8^. Here we provide *in vivo* evidence that independently confirm these earlier findings. Now we add that cellular sumoylation processes are also dysregulated during latent EBV infections by increased *sumo* expression, which increases the intracellular SUMO pools that contribute to the observed increase in protein sumoylation.

Because the cell lines tested would be classified as examples of Type II or Type III EBV latency, it is possible that other EBV latency products, such as LMP2a or LMP2b, can induce *sumo* expression. The finding that cells infected with the EBV mutant P3HR1 still exhibited modestly increased *sumo-1* and *sumo-2/3* levels supports this possibility. To date, EBNA3 is the only one of these three EBV proteins that has been implicated in sumoylation processes due to its ability both to be sumoylated and to interact with sumoylated proteins^28,29,68^. However, the ability of EBNA3 or any other latency-associated proteins to induce *sumo* levels remains untested. The possibility also remains that other EBV latency products or genetic differences between the P3HR1 and WT EBV strains may enhance LMP1-induced *sumo-1* or *sumo-2/3* levels. Regardless, our transient transfection experiments demonstrate that LMP1 is sufficient to induce *sumo* and SUMO expression. These data, along with our previous findings, suggest a role for LMP1 in the increased sumoylation levels detected in latently infected cells.

There was a consistent positive correlation between relative LMP1 RNA levels and sumo levels as well as LMP1 and SUMO levels throughout repeat experiments, and while these results demonstrate that intracellular SUMO pools do change depending on LMP1 expression levels, the correlation between LMP1 and *sumo* or SUMO levels was easier to detect endogenously in EBV-positive cells than in the paired BL41 cells or following transient transfection of 293 cells. We propose that the transformed nature of the HEK 293 cells and the BL41 EBV-negative cells results in increased background *sumo* and SUMO levels, which result in difficulty detecting the even higher *sumo*/SUMO levels induced by LMP1 expression. Serum starvation of the HEK 293 cells reduced endogenous *sumo*/SUMO levels, which allowed the LMP1 dose-dependent increase to be detected. Together, these findings demonstrate that LMP1 is necessary and sufficient for the induction of *sumo-1/2/3* and SUMO-1/2/3 levels during viral latency.

It has been documented that the transcription factor Whn (FoxN1) binds to and activates the *sumo-1* promoter^69^. Additional predicted transcription factor binding sites include Sp1, NF1, Wt1, and E2F^69^. Analysis of the *sumo-1* promoter region^69^, using PROMO, revealed three putative NF-κB binding sites within this region. Findings from experiments using the inhibitor Bay 11–7085 provide the first data demonstrating a role for NF-κB in the induction of *sumo-1*, which supports our proposal that LMP1 induces *sumo-1* levels through canonical NF-κB signaling. To date, the promoter regions for *sumo-2* and *sumo-3* have not been published. However, because we found that Bay 11–7085 treatment of LCLs decreased endogenous *sumo-2/3* and SUMO-2/3 levels, we propose that they will also contain NF-κB binding site(s). In any case, these data are the first to demonstrate that LMP1 induces expression of *sumo* by employing canonical NF-κB signaling, which is activated by CTAR1 and CTAR2. While this is the first report that CTAR1 and CTAR2 have a role in LMP1-induced manipulation of cellular sumoylation processes, it is also the first work to identify a function for NF-κB in the activation of *sumo1/2/3*.

Our earlier report documented that LMP1 induces the covalent modification of proteins by SUMO-1^8^. Here we investigated *sumo* levels in 42 randomly selected B-cell lymphoma samples, which were determined to be either Hodgkin’s lymphoma or non-Hodgkin’s lymphoma (diffuse large B-cell lymphoma). No samples were classified as endemic or non-endemic Burkitt’s lymphoma. Identification of LMP1-positive tissues samples, by real-time PCR, and examination of sectioned tissue by immunofluorescence microscopy revealed LMP1-expressing cells were detected in samples where LMP1 RNA levels were detectable. Specifically, LMP1-expressing cells were detected in both small semi-aggregates and scattered throughout the tissue samples, which led to the conclusion that these samples could be classified as LMP1-positive (or EBV-positive) lymphomas. It is possible that non-malignant, infiltrating, LMP1-positive lymphocytes were the cells detected in the lymphoma tissues; however, we propose that this is not the case due to the high number of LMP1-positive cells detected and the finding that less than 50% of the lymphomas were EBV-positive when significantly more than 50% of patients would have non-malignant, infiltrating, LMP1-positive cells. Regardless, the data did show that the LMP1-positive cells had significantly higher *sumo*/SUMO levels than their LMP1-negative counterparts.

Using immunofluorescence microscopy, we were also able to observe increased endogenous SUMO levels in EBV-positive lymphoma tissues and LMP1-expressing cells. While SUMO was detected in all cells in the tissue samples (LMP1-negative and LMP1-positive samples), SUMO-1 levels were higher in the LMP1-expressing cells, which confirms our earlier findings that demonstrated that LMP1 induces increased levels of total protein sumoylation in cell-culture studies and latently infected cells^8^. Together, these observations are the first to demonstrate that increased levels of SUMO-1 are detected in EBV LMP1-positive lymphoma tissues.

The finding that LMP1-negative lymphoma tissue samples also contained a few cells with increased SUMO-1 levels, suggests that SUMO levels are induced during the process of transformation of B and T cells. Supporting this thought is the finding that over-activation of sumoylation is correlated with a poorer prognosis in multiple myeloma patients^70^. In addition, while SUMO levels were not examined, the increased sumoylation of select target proteins, such as promyelocytic leukemia nuclear bodies, Fas-associated protein with death domain (FADD), and β-catenin^70–72^, has been reported to be critical to the transformation of B and T lymphocytes. Therefore, we propose that all lymphomas will exhibit moderately induced SUMO levels, however, transformation by EBV, and the expression of LMP1, will result in even higher levels of SUMO expression than LMP1-negative lymphomas. Nevertheless, our data here does add EBV-positive B-cell lymphomas to the growing list of malignancies associated with increased SUMO-1 expression^20–26^.

The majority of intracellular SUMO localizes to the nucleus, and the majority of known SUMO targets are nuclear proteins, such as transcription factors, chromatin-associated proteins, and components of the promyelocytic leukemia nuclear bodies. We documented that LMP1 induces the sumoylation of interferon regulatory factor-7 (IRF7) and KRAB-associated protein-1 (KAP1)^7,9^, and LMP1 is known to induce the formation of promyelocytic leukemia nuclear bodies^62,63^, which is tightly regulated by protein sumoylation. We propose that LMP1-induced sumoylation of these select cellular proteins contribute to the observed increased SUMO levels. However, there are almost certainly numerous more targets of LMP1-induced sumoylation. Together, LMP1-induced activation of the *sumo* promoters and LMP1-mediated sumoylation of cellular proteins are responsible for the increased SUMO levels and protein sumoylation detected in latently infected cells and LMP1-positive lymphoma tissues.

Interestingly, increased SUMO levels were also detected in the cytoplasm of EBV-positive lymphoma tissues and LMP1-expressing cells. Traditionally, nuclear proteins are the targets of sumoylation; however, it has been proposed that cytoplasmic and mitochondrial proteins can be targeted by SUMO^73,74^. The cytoplasmic sumoylation processes are proposed to contribute to cell division^75^, modulation of ion channel function^76^, and cellular regulation at the cytoplasmic face of membranes^76^, all of which could aid LMP1-mediated oncogenesis. In addition, oxidative stress, which can occur in lymphomas as well as following EBV-mediated transformation, can result in the cytoplasmic accumulation of SUMO-1^77–79^. Therefore, it would be interesting to identify cytoplasmic targets of LMP1-induced sumoylation as well as the impact of oxidative stress on LMP1-induced sumoylation processes, which would expand our understanding of how LMP1 acts as an oncoprotein.

These results document, for the first time, the correlation between LMP1 expression and SUMO-1 and SUMO-2/3 expression in EBV-infected malignant tissue. They also present a second mechanism by which EBV LMP1 dysregulates cellular sumoylation processes. First, LMP1 hijacks the SUMO-conjugating enzyme^8^, and second, LMP1 activates the *sumo* promoters, increasing the intracellular pools of SUMO. The combined result is increased sumoylation of cellular proteins, which leads to the question if there are additional mechanisms by which LMP1 can regulate cellular sumoylation processes during latent EBV infection.

The findings presented here also expand the list of known malignancies associated with increased SUMO expression and protein sumoylation to include EBV-associated lymphomas. While it has not yet been reported, it is likely that due to their transformed nature most lymphomas have elevated *sumo*/SUMO levels when compared with normal lymphocytes. However, these data demonstrate that LMP1-positive lymphomas have even higher *sumo/*SUMO levels than LMP1-negative lymphomas, which we propose is due to the activation of NF-κB by LMP1 CTAR1 and CTAR2.

Regulation of cellular sumoylation processes is an attractive target for anti-cancer therapies^22,27^. We recently reported that LMP1-induced sumoylation helps stabilize the maintenance of EBV latency^22,27^. Inhibition of sumoylation, by deletion of LMP1 CTAR3 or by treatment with pharmacological inhibitors, resulted in increased sensitivity to viral reactivation and increased susceptibility to anti-viral treatment^22,27^. Therefore, targeting how LMP1 dysregulates cellular sumoylation processes by inhibiting the LMP1-Ubc9 interaction or by regulating the induction of *sumo-1/*2/3 expression may provide a potential method to limit the oncogenesis and progression of EBV-associated lymphomas.

## Electronic supplementary material

Supplemental Info
