# Supplementary material for: Epstein-Barr Virus Latent Membrane Protein-1 Induces the Expression of SUMO-1 and SUMO-2/3 in LMP1-positive Lymphomas and Cells
Source: Sci Rep. 2019 Jan 18;9:208. doi: 10.1038/s41598-018-36312-4 (PMC6338769; doi:10.1038/s41598-018-36312-4)

Epstein-Barr Virus Latent Membrane Protein-1 Induces the Expression of SUMO-1 and SUMO-2/3 in LMP1-positive Lymphomas and Cells.

Sadia Salahuddin, Emma K. Fath, Natalie Biel, Ashley Ray, C. Randall Moss, Akash Patel, Sheetal Patel, Leslie Hilding, Matthew Varn, Tabithia Ross, Wyatt T. Cramblet, Angela Lowrey, Joseph S. Pagano, Julia Shackelford,Gretchen L. Bentz

Unedited blots for Figure 1c, 1d, and 1e: images cropped to dotted-line boxes.

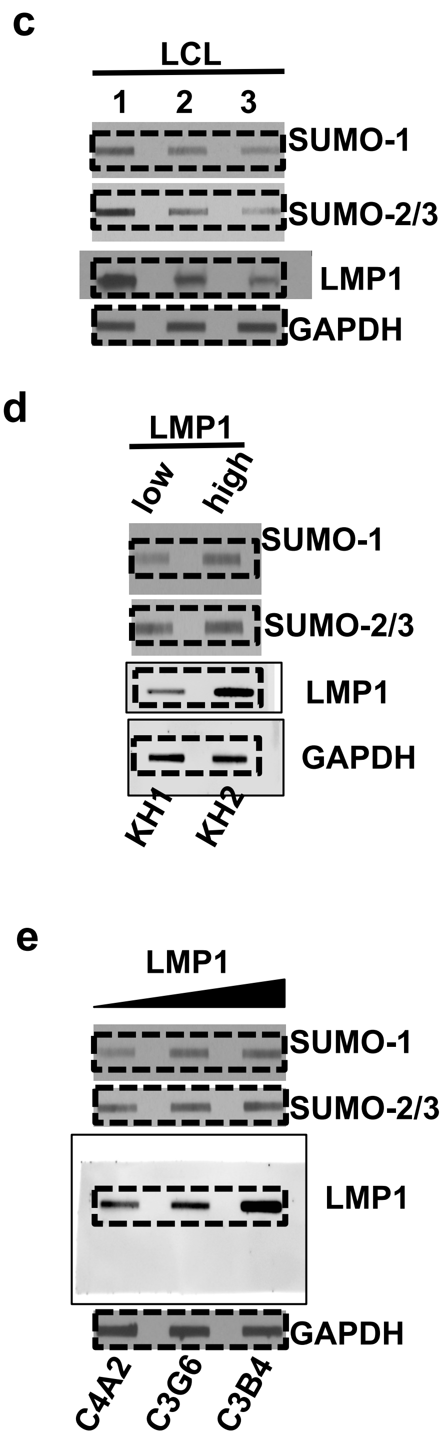

Unedited blots for Figure 2b: images cropped to dotted-line boxes.

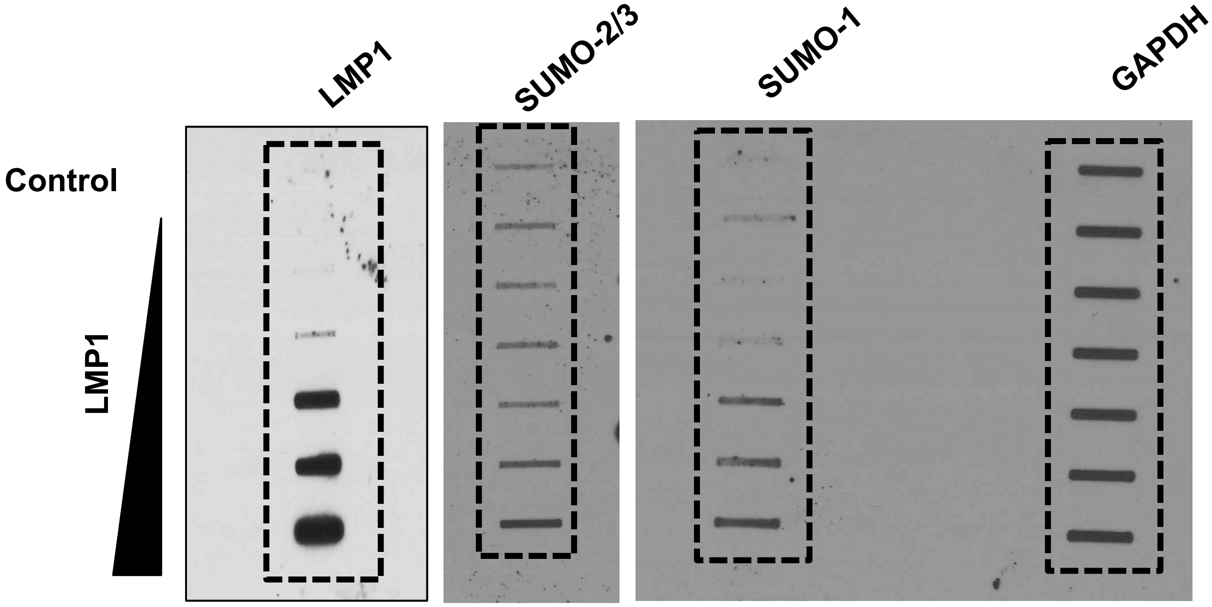

Unedited blots for Figure 3b: images cropped to dotted-line boxes.

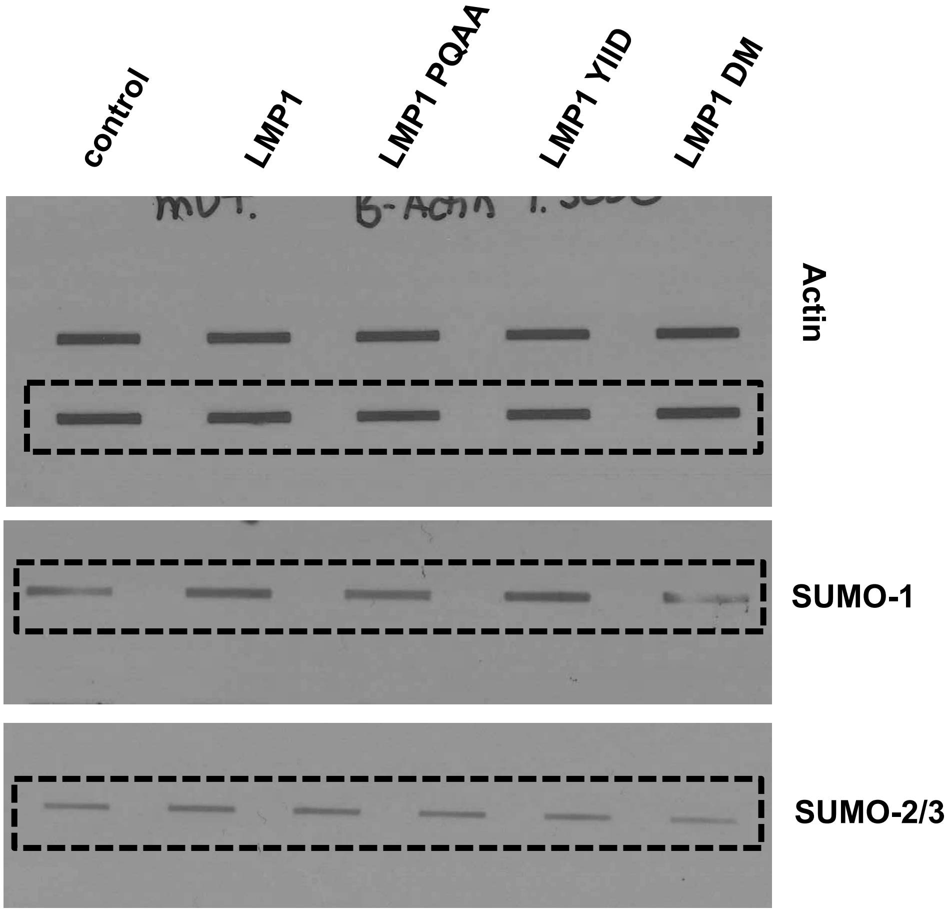

Unedited blots for Figure 3d: images cropped to dotted-line boxes.

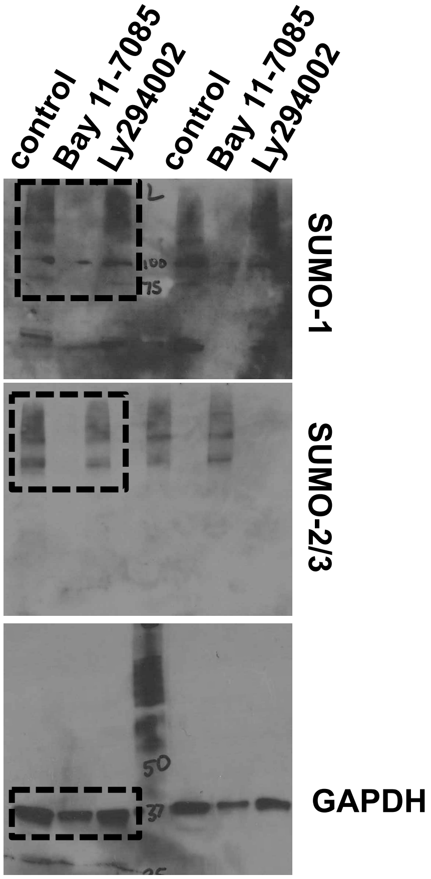

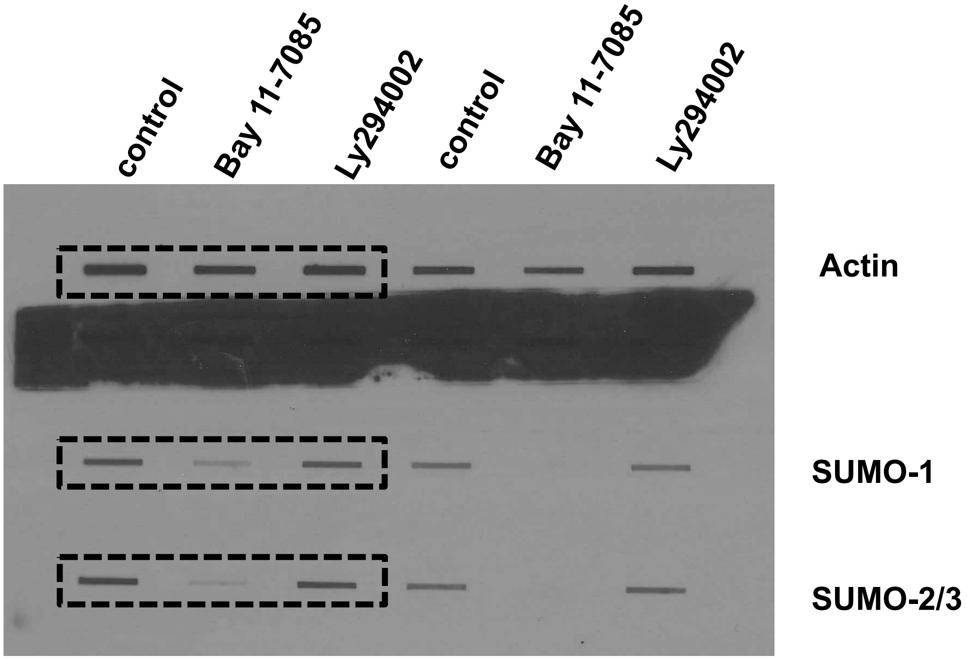

Supplement: Supplementary file 1 — Supplemental Info [file 41598_2018_36312_MOESM1_ESM.pdf]
